# Anti-Protein-Arginine Deiminase 4 IgG and IgA Delineate Severe Rheumatoid Arthritis

**DOI:** 10.3390/diagnostics12092187

**Published:** 2022-09-09

**Authors:** Laura Martinez-Prat, Victor Martinez-Taboada, Cruz Santos, Marcos Lopez-Hoyos, Michael Mahler

**Affiliations:** 1 Research and Development, Autoimmunity, Werfen, OEM Headquarters and Technology Center, 08186 Barcelona, Spain; 2 Department of Rheumatology, Hospital Universitario Marqués de Valdecilla, IDIVAL, 39008 Santander, Spain; 3 Department of Medicine, University of Cantabria, 39005 Santander, Spain; 4 Bioscience Research Institute, Faculty of Experimental Sciences, Universidad Francisco de Vitoria, 28223 Madrid, Spain; 5 Department of Immunology, Hospital Universitario Marqués de Valdecilla, IDIVAL, 39008 Santander, Spain; 6 Molecular Biology Laboratory, Department of Immunology, University of Cantabria, 39005 Santander, Spain; 7 Research and Development, Autoimmunity, Werfen, Autoimmunity Headquarters and Technology Center, San Diego, CA 92131, USA

**Keywords:** rheumatoid arthritis, anti-PAD antibodies, protein-arginine deiminase, PAD, erosion, biologics

## Abstract

There is a strong need for biomarkers of rheumatoid arthritis (RA) in all phases of the patient’s journey and to enable the implementation of precision medicine strategies to improve patient care. The objective of this study was to evaluate the presence of anti-protein-arginine deiminase (PAD) 4 IgG and IgA in the sera of RA patients and disease controls, and to investigate their association with joint erosion and biological treatment use. Sera from 104 RA and 155 controls were tested for the presence of anti-PAD4 IgG and IgA using a new particle-based multi-analyte technology (PMAT). Information on the erosive disease and biological treatment use was available for 54 of the RA patients, who were also tested for anti-citrullinated protein antibodies (ACPA). An association between the autoantibodies and these clinical features was investigated. Anti-PAD4 showed sensitivity and specificity values of 25.0% and 94.2% for IgG and of 21.2% and 94.8% for IgA for RA, respectively. The levels of these antibodies were also significantly higher in RA patients vs. controls, in erosive RA vs. non-erosive disease, and in patients under biologics vs. patients that were not on this treatment regimen. The anti-PAD4 IgG and IgA levels were correlated (rho = 0.60, *p* < 0.0001), but individuals that were positive for only one of the two isotypes were also observed. Anti-PAD4 IgG and IgA are associated with severe RA, and they represent valuable biomarkers for prognosis prediction and patient stratification.

## 1. Introduction

Rheumatoid factor (RF) and anti-citrullinated protein antibodies (ACPA) are valuable markers for the diagnosis of rheumatoid arthritis (RA); nevertheless, there are still a considerable number of patients that are seronegative, especially in early disease [1]. Furthermore, RA-associated joint damage is in general irreversible; therefore, early diagnosis and the initiation of treatment are key factors for therapeutic success. Additionally, RA is a complex heterogeneous disease with variable clinical presentations and manifestations. Due to the disease heterogeneity and complexity, different treatment strategies have been proposed and are used in clinical practice. In patients with more erosive and aggressive forms of disease, treatment escalation to biologics is recommended in the early phase [2]. For these reasons, biomarkers that can help close the serological gap, stratify patients into more homogeneous groups based on clinical and immunological phenotypes, and predict the clinical course and prognosis are warranted, with the end goal being to improve therapeutic decision-making and the facilitation of an appropriate and timely treatment [3]. 

Over the past years, novel serological biomarkers have been described in RA [4]. Some of these markers may not only improve diagnoses by helping to reduce the serological gap, but they might also be helpful during the prognosis and patient stratification processes and in the prediction of responses to different therapeutic approaches. Among them, autoantibodies to the protein-arginine deiminase (PAD) enzymes have shown promise [5]. 

The PAD proteins catalyze the conversion of arginine into citrulline, a post-translational modification known as citrullination that has an important role in normal physiological processes. Citrullination is also very important in the pathogenesis of RA, a disease that is characterized by hypercitrullination, the accumulation of citrullinated products, and the presence of antibodies to the modified antigens [6]. Besides their role in citrullination, the PAD enzymes have also been identified as autoantigens in RA [5,7,8,9], and to date, out of the five family members, IgG antibodies that target PAD4 are the most characterized ones [10]. Several studies have shown that anti-PAD4 IgG represents a useful biomarker to help close the serological gap in RA [11,12]. In addition, variable associations of these antibodies with measures of inflammation, disease activity, or erosion have been repeatedly reported, indicating the clinical value of anti-PAD4 antibodies as prognostic biomarkers that could help stratify patients based on the severity of the disease. Furthermore, associations between anti-PAD4 IgG and ACPA [7], disease severity [9,10,13], worse baseline radiographic joint damage [7], and a better response to treatment escalation [14] have also been reported. 

Despite this association of anti-PAD4 antibodies with disease severity and the independent link between smoking and interstitial lung disease (ILD), little is known about whether there is an etiological association between cigarette smoking and the development of these antibodies, and the data—focused on IgG to date—remain controversial [15,16]. Additionally, autoantibodies to PAD4 have been shown to be present in the pre-clinical phase of RA in a subset of patients. However, the role of anti-PAD4 and the PAD4 enzyme in the development of RA, as well as the timing of the appearance of these antibodies, has not been fully elucidated. 

Notably, ACPA and RF IgA have been reported to be present in pre-clinical RA and to be involved in local mucosal inflammation [17,18]. In particular, circulating ACPA IgA can be found in 30–50% of RA patients [19]; it can be present several years before the disease onset [20], and it has been demonstrated to be linked with cigarette smoking and with more severe disease [21]. Interestingly, it was recently identified as a risk factor for flare ups in combination with ACPA IgG [22]. Although the data are more limited, anti-PAD4 IgG has also been shown to be present in pre-clinical RA [23]. Despite the robust and growing evidence of the utility of anti-PAD4 IgG as an RA biomarker, very little is known about anti-PAD4 IgA. Demoruelle et al. described for the first time the presence of anti-PAD4 IgA in the sera, saliva, and sputum of RA patients, and reported its ability to enhance PAD4’s enzymatic activity [18]. More recently, Darrah et al. investigated the prevalence and clinical significance of different anti-PAD4 isotypes and IgG subclasses, and found that anti-PAD4 IgG1, IgG3, and IgE antibodies identify discrete disease subsets in RA [24]; however, no associations with clinically relevant outcomes were identified in that study. Thus, additional studies that contribute to improving the understanding of the anti-PAD immune response in RA and in pre-clinical disease are needed, and in particular the knowledge around the isotype usage and the implications in the disease manifestations. 

Finally, in a recent study, the immunization of mice with PAD antigens resulted in the development of ACPA and anti-PAD, suggesting that the T-cell response to PAD proteins may trigger ACPAs through a hapten–carrier mechanism [25,26]. Together, these data suggest that anti-PAD antibodies might play a role in RA’s pathogenesis and that they may represent important mechanistic biomarkers to understand the disease course. For these reasons, we aimed to evaluate the presence of anti-PAD4 IgG and IgA in the sera of RA patients and disease controls, and to investigate their association with joint erosion and biological treatment use.

## 2. Materials and Methods

Samples from a total of 259 patients were included in our study, comprising sera from RA patients (*n* = 104) and controls (*n* = 155). The controls included apparently healthy individuals (HI) (*n* = 32), as well as patients with anti-neutrophil cytoplasmic antibody (ANCA)-associated vasculitis (AAV) (*n* = 10), antiphospholipid syndrome (APS) (*n* = 10), celiac disease (CD) (*n* = 10), idiopathic inflammatory myopathies (IIM) (*n* = 10), infectious diseases (ID) (*n* = 20), juvenile idiopathic arthritis (JIA) (*n* = 9), mixed connective tissue disease (MCTD) (*n* = 11), other forms of arthritis (OFA) (*n* = 6), psoriatic arthritis (PsA) (*n* = 8), Sjogren’s syndrome (SjS) (*n* = 7), systemic lupus erythematosus (SLE) (*n* = 11), and systemic sclerosis (SSc) (*n* = 11). The procedures followed were in accordance with the ethical standards of the locally appointed ethics committee and with the Declaration of Helsinki. 

All patient samples were tested using the Aptiva^®^ anti-PAD4 IgG or IgA reagents on the Aptiva^®^ instrument (research use only, RUO). These reagents are based on a particle-based multi-analyte technology (PMAT) that has been described previously [27]. Briefly, a recombinant human full-length PAD4 antigen was covalently bound to the PMAT paramagnetic microparticles. The detection was based on either an anti-human IgG or anti-human IgA antibody conjugated to phycoerythrin. The cut-off used for the anti-PAD4 IgG had been established previously [12], whereas the anti-PAD4 IgA cut-off was established based on the 95th percentile of the controls. Information on the joint erosion status (presence or absence) and biological treatment (yes or no) was available for 54 of the RA patients. The ACPA IgG was also measured in this subset of RA patients with the QUANTA Flash CCP3 chemiluminescence immunoassay (CIA). All tests were from Werfen, San Diego, CA, US.

The data were evaluated using version 5.01 of Analyse-it (Analyse-it Software, Ltd., Leeds, UK). Receiver-operating characteristic (ROC) analyses were carried out to analyze the discrimination between RA patients and controls, and for erosive vs. non-erosive disease types. The sensitivity, specificity, and odds ratio (OR) were calculated based on the preliminary cut-offs. A Wilcoxon–Mann–Whitney analysis was used to analyze differences in titers between groups. A Spearman correlation analysis between the two isotypes was performed. Here, *p*-values < 0.05 were considered significant.

## 3. Results

**Figure 1:**
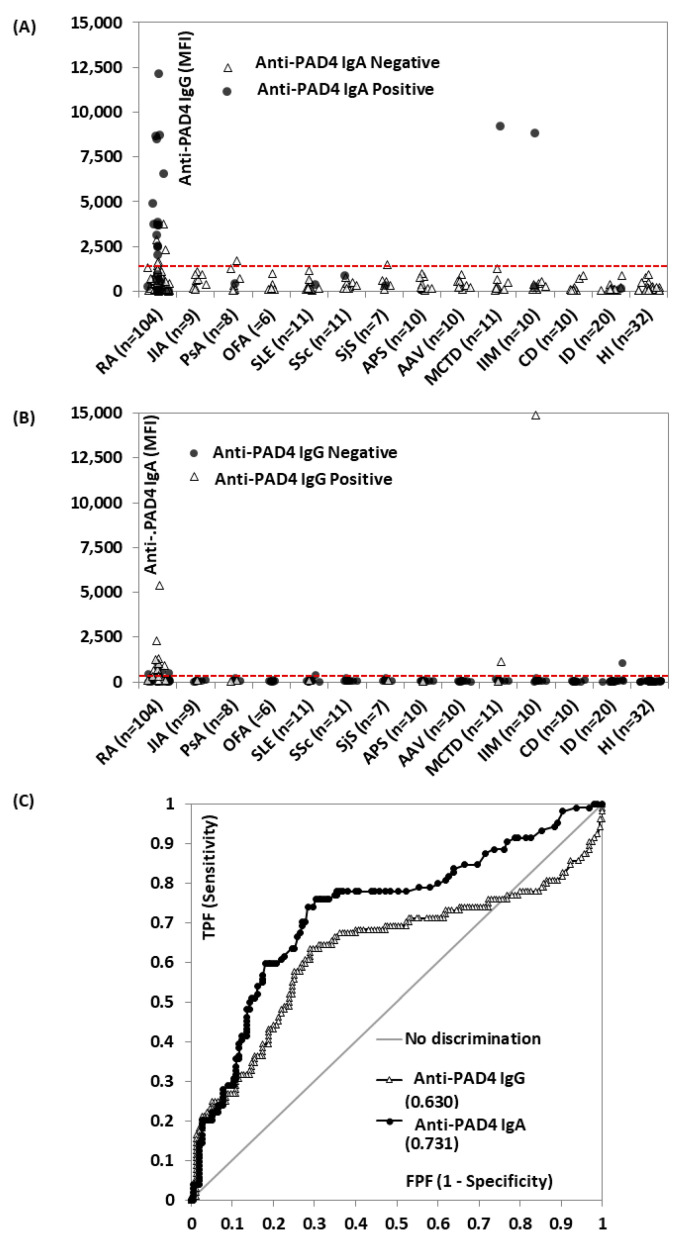
Anti-protein-arginine deiminase (PAD) 4 IgG and IgA in rheumatoid arthritis (RA) patients and controls (*N* = 259). Levels of anti-PAD 4 IgG (**A**) and IgA (**B**) in the different disease groups are shown, labeled by positivity on the other isotype. The red dashed line represents the cut-offs. The receiver operating curve (ROC) analysis of anti-PAD 4 IgG (grey) and IgA (black) for the discrimination of RA patients vs. controls is illustrated in (**C**). The area under the curve (AUC) is shown for each isotype. Abbreviations: AAV: antineutrophil cytoplasmic antibody (ANCA)-associated vasculitis; APS: antiphospholipid syndrome; AUC: area under the curve; CD: celiac disease; HI: healthy individuals; IIM: idiopathic inflammatory myopathies; ID: infections disease; JIA: juvenile idiopathic arthritis; MCTD: mixed connective tissue disease; MFI: median fluorescent intensity; OFA: other forms of arthritis; PAD: protein-arginine deiminase; PsA: psoriatic arthritis; RA: rheumatoid arthritis; ROC: receiver operating characteristic; SjS: Sjogren’s syndrome; SLE: systemic lupus erythematosus; SSc: systemic sclerosis; FPF: false positive fraction; TPF: true positive fraction.

Anti-PAD4 IgG and IgA were observed in 25.0% (26/104) and 21.2% (22/104) of the RA patients, as well as in 5.8% (9/155) and 5.3% (8/155) of the controls, respectively (Figure 1A,B). The prevalence of the double positivity in RA patients was 13.5% (14/104). In the controls, two patients were positive for both isotypes as well, including one with MCTD and one with IIM. The ROC analysis showed significant discrimination between RA patients and controls for both isotypes (Figure 1C). Anti-PAD4 reported sensitivity and specificity values of 25.0% and 94.2% for IgG and 21.2% and 94.8% for IgA, with OR values for RA of 5.4 and 4.9, respectively.

**Figure 2:**
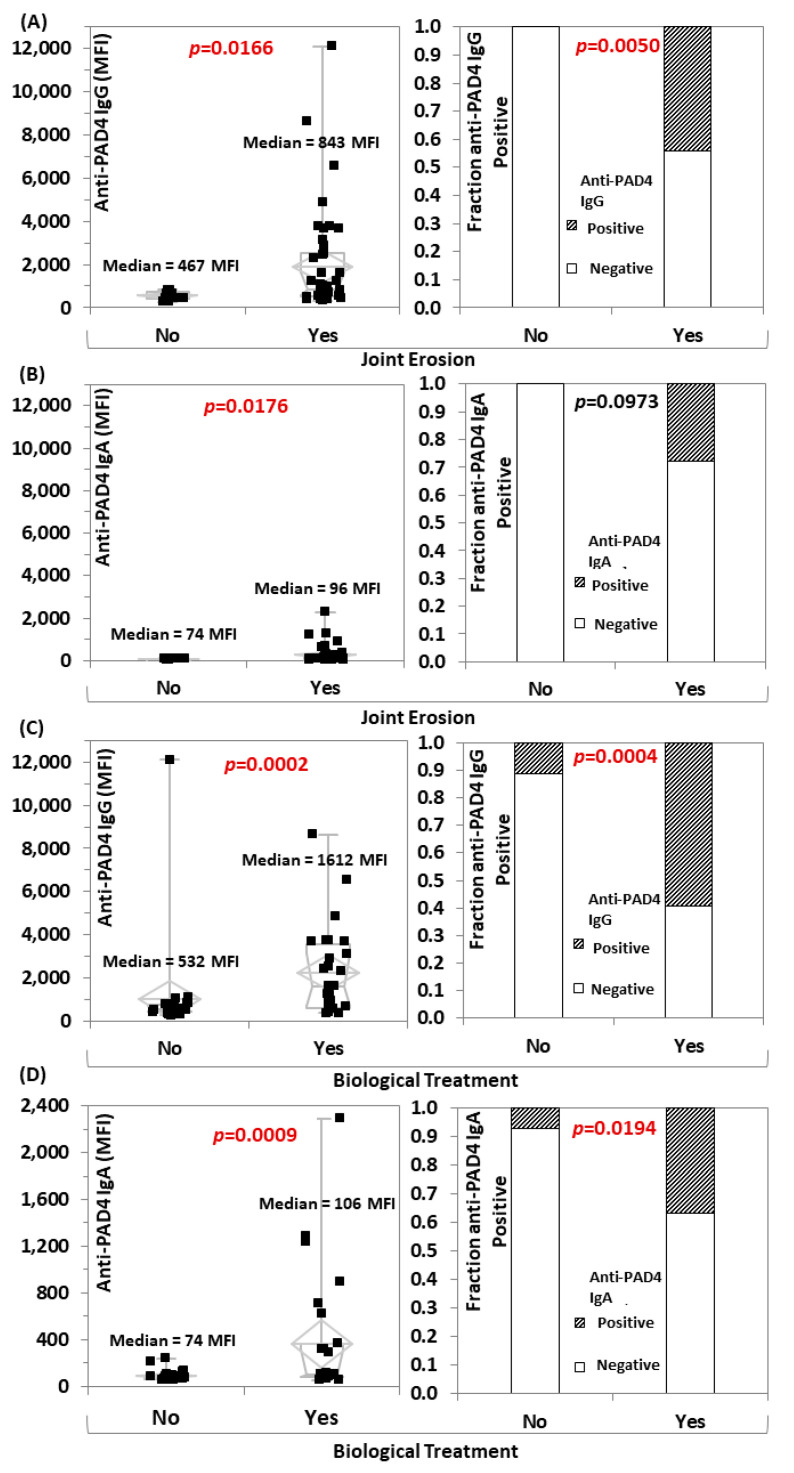
Anti-protein-arginine deiminase (PAD) 4 IgG and IgA are associated with joint erosion and biological treatment use. Pairwise comparison (Wilcoxon–Mann–Whitney analysis) of anti-PAD4 IgG and IgA in RA patients based on erosive status ((**A**,**B**), respectively) and on biological treatment use ((**C**,**D**), respectively) are shown. The median of each subgroup and *p*-values are shown in the figures. Abbreviations: MFI: median fluorescent intensity; PAD: protein-arginine deiminase.

The anti-PAD4 IgG and IgA levels were significantly higher in RA patients vs. controls (*p* = 0.0004 and *p* < 0.0001, respectively) (Figure 2A,B). Interestingly, higher levels of anti-PAD4 IgG and IgA, but not ACPA, were found in the RA patients with erosive disease vs. individuals without erosions (*p* = 0.0166, *p* = 0.0176, and *p* = 0.7883, respectively) (Figure 2A,B), and in patients under biological treatment vs. those that were not on biologics (*p* = 0.0002, *p* = 0.0009, and *p* = 0.7752, respectively) (Figure 2C,D).

**Figure 3:**
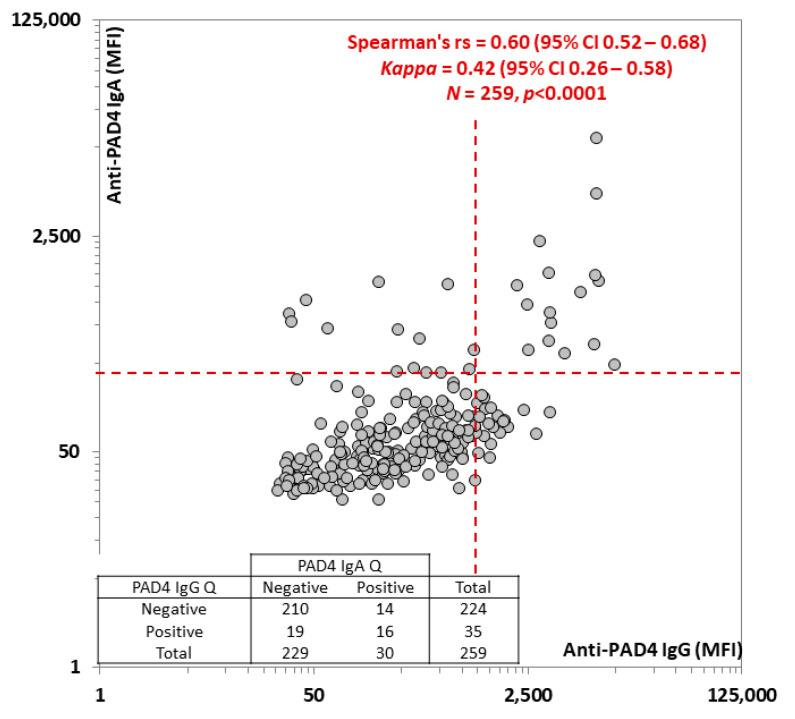
Correlation between anti-protein-arginine deiminase (PAD) 4 IgG and IgA levels. The Spearman correlation analysis between anti-PAD4 IgG and IgA is shown. The red dashed line represents the cut-offs. Spearman’s rs, the kappa coefficient, number of patients included, and *p*-values are shown in the graph. The qualitative agreement is shown in the table. Abbreviations: MFI: median fluorescent intensity; PAD: protein-arginine deiminase; Q: qualitative.

Although the anti-PAD4 IgG and IgA levels were correlated (rho = 0.60, *p* < 0.0001), and a significant portion of the patients expressed both isoforms, several patients were positive for only one of the two isotypes (overlap and correlation are shown in Figure 3).

**Figure 4:**
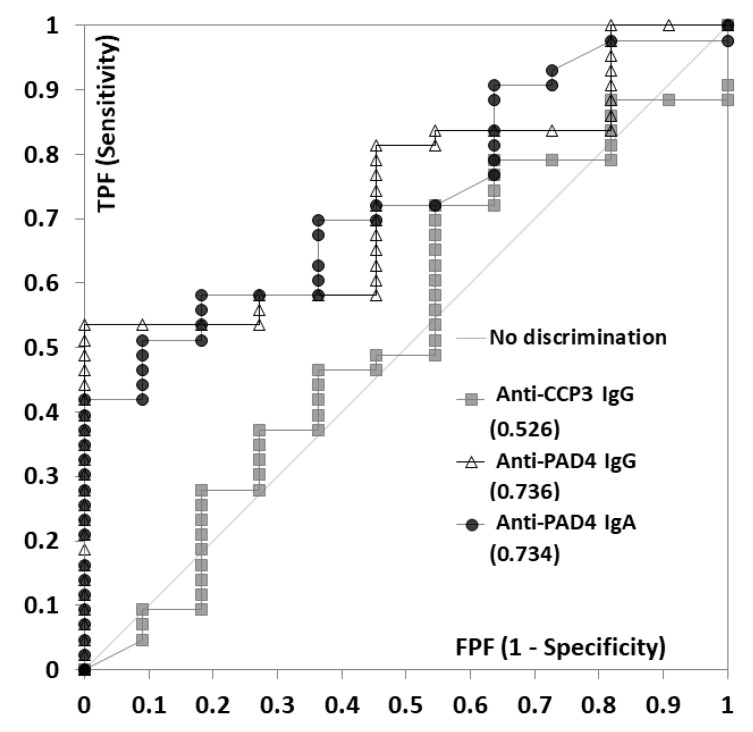
Anti-protein-arginine deiminase (PAD) 4 IgG and IgA and anti-cyclic citrullinated peptide (CCP) 3 in erosive rheumatoid arthritis (RA) (*n* = 54). The receiver operating curve (ROC) analysis of anti-PAD 4 IgG (triangles) and IgA (circles), and CCP3 (squares) for the discrimination of RA erosive disease versus the absence of this manifestation. The area under the curve (AUC) is shown for each biomarker. Abbreviations: CCP: cyclic citrullinated peptide; PAD: protein-arginine deiminase; FPF: false positive fraction; TPF: true positive fraction.

In the discrimination of erosive from non-erosive diseases, the ROC analysis showed higher area under the curve values for anti-PAD4 IgA (0.736), followed by IgG (0.734) and ACPA (0.636) (Figure 4). All patients that were positive for anti-PAD4 IgG or IgA, or both isotypes, had erosive disease. The anti-PAD4 IgG and IgA positive patients were 11.6 (95% CI 2.9–45.6) and 7.4 (95% CI 1.5–33.7) times more likely to be on biologics compared to the negative group. Patients that were double positive for both isotypes were 13.0 (95% CI 1.9–85.9) times more likely to be on biologics.

## 4. Discussion

Historically, RF- and ACPA-seropositive RA has been known to represent a more aggressive disease phenotype [28,29]. However, this paradigm might be changing due to the better understanding and management of ACPA-positive RA [30]. Consequently, novel markers that help to stratify patients based on disease phenotypes are important to further improve the prognosis prediction and management of RA. Anti-PAD4 IgG has been reported to be associated with ACPA and a more severe disease course in several independent studies [7,9,13]. Anti-PAD4 IgG has also been demonstrated to be present in ACPA negative individuals [12]. In our study, the association between anti-PAD4 IgG and erosive and severe disease—associated with the use of biologics—was confirmed. However, interestingly, this association was not observed for ACPA. Unfortunately, the erosion and disease activity scores and the results for inflammation markers were not available for our RA samples, meaning their potential correlation with anti-PAD4 antibodies could not be interrogated in our study. Despite this constraint, these results indicate that anti-PAD4 antibodies represent useful biomarkers to help predict disease severity complementary to ACPA. Therefore, anti-PAD4 could contribute to improving the management of RA patients and could be used to support treatment decisions.

In this study, we have confirmed the presence of anti-PAD4 IgA in the serum samples of individuals with RA, as previously reported by Demoruelle et al. [18] and Darrah et al. [24]. Importantly, we have been the first to evaluate this anti-PAD4 isotype in a relatively large set of patients that included a high number of disease controls. Remarkably, in our cohort, anti-PAD4 IgA reported high specificity. While a moderate correlation between the two anti-PAD4 isotypes was observed (rho = 0.60, *p* < 0.0001), patients positive for only one of them were also identified. Whether this corresponds to patients in different disease stages or different disease phenotypes beyond erosion could not be investigated in our study. The timing of the appearance of these antibodies, their evolution with the disease progression, and whether the antibody levels could change with treatment remain areas of study, especially with regards to autoantibodies to the PAD enzymes. One additional important aspect to consider is that it is unknown whether different treatments could have differential effects on the antibody levels. One limitation of this study was that the information on the specific treatment for each patient was not available; therefore, we could not interrogate whether there could be differences between therapies based on different therapeutic targets or mechanisms of action.

Recently, Darrah et al. studied the isotype usage of the B-cell immune response to PAD4 and reported the highest prevalence for IgG1 (28.6%), followed by IgG3 (25.5%), IgG4 (25.5%), IgE (25.0%), IgG2 (21.2%), IgA (20.9%), and IgM (9.2%), all adjusted to 95% specificity [24]. The prevalence levels for anti-PAD4 IgA in RA were almost identical between our current investigation and the studies by Darrah et al. Interestingly, in the latest study, anti-PAD4 IgA antibodies were strongly associated with anti-PAD3/4 cross-reactivity antibodies, an antibody population that is associated with erosive RA [7]. However, contradictory to our study, anti-PAD4 IgA was not associated with joint erosions, or with any of the clinically relevant outcomes that were interrogated in the study. The difference in the results between Darrah et al.’s study and our work presented here might be explained by the different methods applied. While Darrah et al. utilized an ELISA, in our study we measured anti-PAD4 antibodies using PMAT. In addition, the difference might also be attributed to the specificity of the anti-human IgA conjugate. More specifically, it has been demonstrated that IgA1 and IgA2 have different properties, with IgA2 exhibiting pro-inflammatory properties linked to glycosylation [31,32,33]. Further studies are needed to analyze the differences between Darrah et al.’s results and our findings, in order to continue to understand the anti-PAD4 response and isotype usage and to further elucidate the utility of anti-PAD4 IgA as a biomarker in RA.

Several studies have demonstrated a link between the development of mucosal dysbiosis, inflammation, and autoantibody production, and the next stages of development of systemic autoimmunity [17]. In this context, the presence of ACPA IgA has been reported in pre-clinical RA and established RA, and the local and systemic presence of these antibodies seems to be associated with local mucosal inflammation [34]. Unfortunately, a potential association between anti-PAD4 IgA and mucosal inflammation or with other potentially associated relevant clinical features such as pulmonary manifestations, including ILD, could not be assessed in our study due to the lack of clinical information in this direction. It will be interesting to investigate in future studies the clinical significance of the anti-PAD4 IgA isotype in RA and whether its presence is associated with different disease stages, or even a distinct underlying pathogenic mechanism.

## 5. Conclusions

In conclusion, our study confirms the presence of anti-PAD4 IgA in the serum samples of RA patients and the association of anti-PAD4 IgG with erosive RA, and is the first to report anti-PAD4 IgA as a highly specific marker for RA. A strong association between anti-PAD4 IgG and IgA and erosive disease and biological treatment was observed, indicating that these biomarkers could represent important tools for patient stratification in RA.

## 6. Patents

Patents resulting from the work reported in this manuscript (all pending): 16/791779 (US); 3129624 (Canada); 2020221375 (Australia); 2021547276 (Japan); 3924736 (Europe).

## Data Availability

Not applicable.

## Abbreviations

AAV: antineutrophil cytoplasmic antibody (ANCA)-associated vasculitis; ACPA: anti-citrullinated peptide/protein antibodies; APS: antiphospholipid syndrome; AUC: Area under the curve; CCP: cyclic citrullinated peptide; CD: celiac disease; HI: healthy individuals; IIM: idiopathic inflammatory myopathies; ILD: interstitial lung disease, ID: infections disease; JIA: juvenile idiopathic arthritis; MCTD: mixed connective tissue disease; MFI: median fluorescent intensity; OFA: other forms of arthritis; PAD: protein-arginine deiminase; PsA: psoriatic arthritis; RA: rheumatoid arthritis; ROC: receiver operating characteristic; SjS: Sjogren’s syndrome; SLE: systemic lupus erythematosus; SSc: systemic sclerosis.

## References

[1] Trouw L., Mahler M. (2012). Closing the serological gap: Promising novel biomarkers for the early diagnosis of rheumatoid arthritis. Autoimmun. Rev..

[2] Demoruelle M.K., Deane K.D. (2012). Treatment strategies in early rheumatoid arthritis and prevention of rheumatoid arthritis. Curr. Rheumatol. Rep..

[3] Smolen J.S., Aletaha D., Barton A., Burmester G.R., Emery P., Firestein G.S., Kavanaugh A., McInnes I.B., Solomon D.H., Strand V. (2018). Rheumatoid arthritis. Nat. Rev. Dis. Primers.

[4] Van Delft M.A., Huizinga T.W. (2020). An overview of autoantibodies in rheumatoid arthritis. J. Autoimmun..

[5] Curran A.M., Naik P., Giles J.T., Darrah E. (2020). PAD enzymes in rheumatoid arthritis: Pathogenic effectors and autoimmune targets. Nat. Rev. Rheumatol..

[6] Darrah E., Andrade F. (2018). Rheumatoid arthritis and citrullination. Curr. Opin. Rheumatol..

[7] Darrah E., Giles J.T., Ols M.L., Bull H.G., Andrade F., Rosen A. (2013). Erosive Rheumatoid Arthritis Is Associated with Antibodies That Activate PAD4 by Increasing Calcium Sensitivity. Sci. Transl. Med..

[8] Darrah E., Giles J.T., Davis R.L., Naik P., Wang H., Konig M.F., Cappelli L.C., Bingham C.O.I., Danoff S.K., Andrade F. (2018). Autoantibodies to peptidylarginine deiminase 2 are associated with less severe disease in rheumatoid arthritis. Front. Immunol..

[9] Halvorsen E.H., Pollmann S., Gilboe I.-M., van der Heijde D., Landewe R., Odegard S., Kvien T.K., Molberg O. (2008). Serum IgG antibodies to peptidylarginine deiminase 4 in rheumatoid arthritis and associations with disease severity. Ann. Rheum. Dis..

[10] Martinez-Prat L., Palterer B., Vitiello G., Parronchi P., Robinson W.H., Mahler M. (2019). Autoantibodies to protein-arginine deiminase (PAD) 4 in rheumatoid arthritis: Immunological and clinical significance, and potential for precision medicine. Expert Rev. Clin. Immunol..

[11] Ren J., Sun L., Zhao J. (2017). Meta-analysis: Diagnostic accuracy of antibody against peptidylarginine deiminase 4 by ELISA for rheumatoid arthritis. Clin. Rheumatol..

[12] Martinez-Prat L., Lucia D., Ibarra C., Mahler M., Dervieux T. (2019). Antibodies targeting protein-arginine deiminase 4 (PAD4) demonstrate diagnostic value in rheumatoid arthritis. Ann. Rheum. Dis..

[13] Darrah E., Martinez-Prat L., Mahler M. (2019). Clinical utility of antipeptidyl arginine deiminase type 4 antibodies. J. Rheumatol..

[14] Darrah E., Yu F., Cappelli L.C., Rosen A., O’Dell J.R., Mikuls T.R. (2019). Association of baseline peptidylarginine deiminase 4 autoantibodies with favorable response to treatment escalation in rheumatoid arthritis. Arthritis Rheumatol..

[15] Cappelli L.C., Konig M.F., Gelber A.C., Iii C.O.B., Darrah E. (2018). Smoking is not linked to the development of anti-peptidylarginine deiminase 4 autoantibodies in rheumatoid arthritis. Arthritis Res. Ther..

[16] Giles J.T., Darrah E., Danoff S., Johnson C., Andrade F., Rosen A., Bathon J.M. (2014). Association of cross-reactive antibodies targeting peptidyl-arginine deiminase 3 and 4 with rheumatoid arthritis-associated interstitial lung disease. PLoS ONE.

[17] Holers V.M., Demoruelle M.K., Kuhn K.A., Buckner J.H., Robinson W.H., Okamoto Y., Norris J.M., Deane K.D. (2018). Rheumatoid arthritis and the mucosal origins hypothesis: Protection turns to destruction. Nat. Rev. Rheumatol..

[18] DDemoruelle M.K., Wang H., Davis R.L., Visser A., Hoang J., Norris J.M., Holers V.M., Deane K.D., Darrah E. (2021). Anti-peptidylarginine deiminase-4 antibodies at mucosal sites can activate peptidylarginine deiminase-4 enzyme activity in rheumatoid arthritis. Arthritis Res. Ther..

[19] Lakos G., Soós L., Fekete A., Szabó Z., Zeher M., Horváth I.F., Dankó K., Kapitány A., Gyetvai A., Szegedi G. (2008). Anti-cyclic citrullinated peptide antibody isotypes in rheumatoid arthritis: Association with disease duration, rheumatoid factor production and the presence of shared epitope. Clin. Exp. Rheumatol..

[20] Kelmenson L.B., Wagner B.D., McNair B.K., Frazer-Abel A., Demoruelle M.K., Bergstedt D.T., Feser M.L., Moss L.K., Parish M.C., Mewshaw E.A. (2020). Timing of elevations of autoantibody isotypes prior to diagnosis of rheumatoid arthritis. Arthritis Rheumatol..

[21] Svärd A., Kastbom A., Reckner-Olsson Å., Skogh T. (2008). Presence and utility of IgA-class antibodies to cyclic citrullinated peptides in early rheumatoid arthritis: The Swedish TIRA project. Arthritis Res. Ther..

[22] Sokolova M.V., Hagen M., Bang H., Schett G., Rech J., Steffen U., Haschka J., Englbrecht M., Hueber A.J., Manger B. (2022). IgA anti-citrullinated protein antibodies are associated with flares during DMARD tapering in rheumatoid arthritis. Rheumatology.

[23] Kolfenbach J.R., Deane K.D., Derber L.A., O’Donnell C.I., Gilliland W.R., Edison J.D., Rosen A., Darrah E., Norris J.M., Holers V.M. (2010). Autoimmunity to peptidyl arginine deiminase type 4 precedes clinical onset of rheumatoid arthritis. Arthritis Care Res..

[24] Gómez-Bañuelos E., Shi J., Wang H., Danila M., Bridges S., Giles J., Sims G., Andrade F., Darrah E. (2022). Heavy chain constant region usage in antibodies to peptidylarginine deiminase 4 distinguishes disease subsets in rheumatoid arthritis. Arthritis Rheumatol..

[25] Arnoux F., Mariot C., Peen E., Lambert N.C., Balandraud N., Roudier J., Auger I. (2017). Peptidyl arginine deiminase immunization induces anticitrullinated protein antibodies in mice with particular MHC types. Proc. Natl. Acad. Sci. USA.

[26] Auger I., Balandraud N., Massy E., Hemon M.F., Peen E., Arnoux F., Mariot C., Martin M., Lafforgue P., Busnel J. (2020). Peptidylarginine deiminase autoimmunity and the development of anti–citrullinated protein antibody in rheumatoid arthritis: The hapten–carrier model. Arthritis Rheumatol..

[27] Richards M., LA Torre I.G.-D., González-Bello Y.C., Mercado M.V.-D., Andrade-Ortega L., Medrano-Ramírez G., Navarro-Zarza J.E., Maradiaga-Ceceña M., Loyo E., Rojo-Mejía A. (2019). Autoantibodies to Mi-2 alpha and Mi-2 beta in patients with idiopathic inflammatory myopathy. Rheumatology.

[28] Hecht C., Englbrecht M., Rech J., Schmidt S., Araujo E., Engelke K., Finzel S., Schett G. (2015). Additive effect of anti-citrullinated protein antibodies and rheumatoid factor on bone erosions in patients with RA. Ann. Rheum. Dis..

[29] Van Steenbergen H.W., Ajeganova S., Forslind K., Svensson B., Van Der Helm-van Mil A.H.M. (2015). The effects of rheumatoid factor and anticitrullinated peptide antibodies on bone erosions in rheumatoid arthritis. Ann. Rheum. Dis..

[30] Boer A.C., Boonen A., van der Helm van Mil A.H. (2018). Is Anti-citrullinated protein antibody-positive rheumatoid arthritis still a more severe disease than anti-citrullinated protein antibody-negative rheumatoid arthritis? A Longitudinal cohort study in rheumatoid arthritis patients diagnosed From 2000 onward. Arthritis Care Res..

[31] Delacroix D., Van Snick J., Vaerman J., Conley M., Mascart-Lemone F., Bernier G. (1986). Monoclonal antibodies against isotypic and isoallotypic determinants of human IgA1 and IgA2: Fine specificities and binding properties. Mol. Immunol..

[32] Boackle R.J., Nguyen Q.L., Leite R.S., Yang X., Vesely J. (2006). Complement-coated antibody-transfer (CCAT); serum IgA1 antibodies intercept and transport C4 and C3 fragments and preserve IgG1 deployment (PGD). Mol. Immunol..

[33] Steffen U., Koeleman C.A., Sokolova M.V., Bang H., Kleyer A., Rech J., Unterweger H., Schicht M., Garreis F., Hahn J. (2020). IgA subclasses have different effector functions associated with distinct glycosylation profiles. Nat. Commun..

[34] Demoruelle M.K., Harrall K.K., Ho L., Purmalek M.M., Seto N.L., Rothfuss H.M., Weisman M.H., Solomon J.J., Fischer A., Okamoto Y. (2017). Anti–citrullinated protein antibodies are associated with neutrophil extracellular traps in the sputum in relatives of rheumatoid arthritis patients. Arthritis Rheumatol..

